# Comparative analysis of knowledge, attitudes, and breast cancer screening practices among women in South Africa and the United States: a multinational study

**DOI:** 10.21203/rs.3.rs-9151807/v1

**Published:** 2026-04-25

**Authors:** Monica Ewomazino Akokuwebe, Wenlong Carl Chen, Raylton Chikwati, Rebaone Petlele, Olaide Ojoniyi, Daniel S O’Neil, Shane Norris, Maureen Joffe

**Affiliations:** SAMRC Developmental Pathways for Health Research Unit, Department of Paediatrics, University of the Witwatersrand; SAMRC Developmental Pathways for Health Research Unit, Department of Paediatrics, University of the Witwatersrand; SAMRC Developmental Pathways for Health Research Unit, Department of Paediatrics, University of the Witwatersrand; SAMRC Developmental Pathways for Health Research Unit, Department of Paediatrics, University of the Witwatersrand; SAMRC Developmental Pathways for Health Research Unit, Department of Paediatrics, University of the Witwatersrand; Yale Cancer Center, Yale University, and Yale School of Medicine, Yale University; SAMRC Developmental Pathways for Health Research Unit, Department of Paediatrics, University of the Witwatersrand; SAMRC Developmental Pathways for Health Research Unit, Department of Paediatrics, University of the Witwatersrand

**Keywords:** Breast cancer screening, Knowledge, Attitudes, and Practices, South Africa, United States

## Abstract

**Background:**

Breast cancer screening is an effective prevention strategy, yet global disparities in uptake persist across and within countries from varying human development index contexts. This study compares breast cancer screening-related knowledge, attitudes, and practices among US and SA women.

**Methods:**

Cross-sectional studies were conducted among 2000 age-matched SA and US women. Sociodemographic data, knowledge, attitudes, and screening practice, were assessed. Multivariate logistic regression associations between screening behaviors (ever screened, clinical breast examination (CBE), mammography) and predictors were determined.

**Results:**

US women had greater breast cancer knowledge and were more likely to participate in screening (ever screened AOR = 2.078, p < 0.001, CBE AOR = 2.741, p < 0.001, and mammography AOR = 3.712, p < 0.001). SA women perceived breast cancer to be less serious than US women, but perceived greater benefits and self-efficacy for breast self-examination and greater cues to action. Screening uptake was mainly associated with availability of screening services; knowledge, family history of breast cancer, older age, tertiary education, marital status and good health also contributed to increased screening behavior. Unexpectedly, stigma associated with increased perceived benefits of screening and screening practice and self-efficacy for BSE and cues to action.

**Conclusions:**

Tailored interventions should incorporate structural investments and stigma-sensitive education to increase screening participation. SA should prioritize increasing community knowledge and expanding CBE into communities and primary health care. US policies should focus on marginalized minority groups through insurance expansion, navigation support, and tailored outreach.

## Introduction

In 2022, approximately 2,296,840 new breast cancer (BC) cases (representing a quarter of all cancer cases among women) and 670,000 breast cancer related deaths occurred (15.5% of total cancer fatalities)[1, 2]. With greater life expectancy and prevalence of lifestyle risk factors, high-income countries (HICs) exhibit the highest BC incidence (age-standardized incidence rates (ASIRs) at 80 to >100 per 100,000 population, approximately double that of low- and middle-income countries (LMICs), with ASIRs from 28 to >55[3–5]. LMICs already contribute 53% of all new BC cases and 62% of breast cancer mortality globally, and with increasing life expectancy and lifestyle transitions, incidence is rapidly rising [6–8]. In HICs, age-standardized mortality rates (ASMRs), currently at 12.32 for North America in 2022[5], have declined by 40% between 1980 and 2020. This is due to population-based mammography screening and state-of-the-art diagnostic and treatment services. In contrast, in many LMICs, ASMRs for BC can reach as high as 20-30 per 100,000 women, and mortality-to-incidence ratios significantly exceed those of HICs[3, 4]. This high mortality rate is associated with comparatively lower community knowledge and awareness of BC, a lack of population-based screening, inadequate diagnostic and treatment resources, and unaffordability of services in countries where universal access is not provided[9]. LMIC patients are consequently diagnosed with predominantly late-stage disease, receive inadequate treatments, and suffer high mortality rates.

South Africa (SA), an upper-middle-income country, has a rapidly increasing cancer burden. BC accounts for 22.6% of female cancers and 16% of their cancer deaths [8, 10]. The 2022 SA National Cancer Registry (SANCR) reported ASIR at 94.56 per 100,000 for white women, 50.86 per 100,000 for Asian women, 47.47 per 100,000 for women of mixed ancestry, and 26.08 per 100,000 for black women[11] Some 60% of patients accessing the SA public health services present with late-stage (III or IV) disease[12]. They are also burdened with high prevalences of human immunodeficiency virus (HIV), hypertension, and obesity[6]. We reported 5-year crude overall survival (OS) to be just 44.3% (95% Confidence Interval (CI) 42.5-46.2)[13].

Poverty is frequently associated with lower access to screening, diagnostic, and treatment services, poorer knowledge and awareness of breast cancer, attitudes marked by fear, perceived low susceptibility, and mistrust of healthcare systems[14–19]. Addressing these challenges requires multifaceted approaches to improve BC knowledge, increase access to screening services, and address socioeconomic and cultural barriers to screening service uptake[20–22]. A knowledge, attitudes, and practice (KAP) study across diverse economic groups and varying access to screening services can provide policymakers with insights into barriers to preventative BC behaviors. Using a conceptual model (Figure 1) integrating the KAP framework and Health Belief Model (HBM)[23–26], this study: i) evaluated and compared BC screening practices among women in the US and SA, and ii) examined how knowledge and attitudes shaped screening behaviors.

## Methods

### Study setting and population characteristics

The US is a high human development index (HDI) country with a total gross national income (GNI) of US$26.9 trillion and a per capita GNI of US$80,450 in 2023[27]. In 2024, 64.4% of the population under 65 years of age has access to private health insurance, and an additional 28.6% has access to public health insurance programs[28, 29]. Private health systems offer quick access to high-quality medical services but are expensive, leading to health inequalities[30]. Public health insurance covers primary health care and BC screening through Medicare, Medicaid, and Veterans Health Administration)[31]. However, some minority groups remain uninsured, and experience lower BC screening uptake, diagnostic, and treatment services[31, 32].

SA has an estimated population of 63.2 million people in 2025, with a GNI per capita of about US$6,480 and is the second largest economy on the African continent[19, 22]. The majority (84%) of the population is impoverished, depending on the resource-constrained public health care system[33], with limited mammography screening services. Nevertheless, unlike in many other sub-Saharan African (SSA) countries, public cancer diagnostic and treatment services are provided free to patients, who do bear minimal out-of-pocket transport and hospital access costs. The remaining 16% of higher income citizens receive health services including mammography screening, from the private sector via private health insurance and out-of-pocket contributions[32].

### Study design and participants

This study used cross-sectional questionnaire data collected from 2,000 participants from SA and the US (n = 1,000 per country). Participants were women aged 35–70 years recruited through the IPSOS i-Say panel[34] and surveyed in May 2025. Proportional recruitment was applied: 20% aged 35–44, 40% each aged 45–54 and 55–70 years. The IPSOS i-Say panel is an international online population panel with diverse participants who consent to be contacted for surveys. Eligible members from SA and the US were invited, consented, and completed our custom KAP survey. Respondents were verified through the IPSOS i-Say system, and de-identified data were provided for analysis. The KAP survey included knowledge, attitude, and practice components from validated instruments including the Modified Thai version of the Champion’s Health Belief Model Scale (MT-CHBMS)[16, 17, 19, 20, 24–26, 35–43]. Internal consistency for HBM[43–45] and KAP[25, 36, 38, 39, 41–43] questionnaires has been previously confirmed. Each country’s questionnaire comprised 48 items, covering socio-demographics, with socioeconomic status measured by living standard measure (LSM) in SA and annual household income in the US[28, 30, 46, 47]. BC screening practices (ever screened, clinical breast examination, mammography) were assessed. Questionnaires were adapted for cultural context. KAP scores were aggregated using binary coding (Correct = 1, Incorrect = 0) and 5-point Likert scales (1 = strongly disagree to 5 = strongly agree). Surveys are provided as Supplementary Documents Appendix A and B. Ordered constructs were coded consistently so that higher values reflected higher levels of the underlying construct.

### Modeling and statistical analysis approaches

Descriptive statistics were used to summarize participant characteristics, knowledge, HBM constructs, health system access variables, and screening behaviors. Demographics and covariate factors included age group, education level, employment status, wealth score, marital status, parity, family history of breast cancer, self-rated health, mental health status, country of residence, availability of CBE services, availability of mammography services, and perceived community stigma related to breast cancer. Reference categories were selected to reflect the lowest level of exposure or risk. Categorical variables were summarized using frequencies and percentages.

All regression analyses followed a pre-specified, theory-driven three-stage modelling approach, aligned with the conceptual model (figure 1), in which knowledge and attitudes (HBM individual perception variables), as sequential intermediate outcomes, precede screening behavior. This approach allowed for separation of cognitive, attitudinal, and behavioral determinants while maintaining interpretability of effect estimates. Across all models, results are reported as adjusted odds ratios with 95% confidence intervals. Statistical significance was assessed at the 5% level, with borderline associations interpreted cautiously in the context of effect size, consistency across models, and biological or behavioral plausibility

Ordinal logistic regression (ologit) models were used to evaluate associations with higher versus lower interim outcome categories (*i.e.,* knowledge and attitude outcomes). Because the proportional odds assumption may not always hold for attitudinal constructs, generalized ordered logistic regression (gologit2) models with autofit were also estimated. When assumptions were not violated, standard ordinal logit estimates were retained for interpretability and reported as primary estimates, with generalized models used to confirm robustness of direction and magnitude of effects. Where violations were detected, generalized estimates were used to confirm stability of associations. For ordinal models, proportional odds assumptions were evaluated using Wald tests within the generalized ordered logistic framework

Binary screening behavioral outcomes (*i.e.,* behavior outcomes) were analyzed using multivariable logistic regression, with knowledge, all HBM attitudinal constructs, socio-demographic variables, and health system access factors included simultaneously. Overall model fit was assessed using likelihood ratio chi-square tests, pseudo R^2^ statistics, and log likelihood values. Complete-case analysis was used for all models and analyses were conducted using Stata 17, with ologit, gologit2, and logit commands used for ordered and binary outcomes as appropriate.

## Results

### Characteristics of participants

As shown in Table 1, more US women (51% of those willing to declare their income) earned less than $50,000 and were from lower relative income strata compared with 33% in SA. More US women were unemployed, had no children, had lower perceived health status, and had greater breast cancer knowledge and access to mammography screening services than SA women.

### Factors associated with breast cancer knowledge

From the generalized ordered logistic regression analysis (Table 2), SA women had lower BC knowledge than US women (AOR=0.50, 95% CI: 0.40-0.63, p<0.001). Higher BC knowledge was associated with tertiary education (AOR =1.32, 95% CI: 1.08–1.62), being divorced (AOR =1.46, 95% CI: 1.08–1.99, p=0.015), mild/moderate mental health symptoms (AOR =1.36, 95% CI: 1.10–1.69, p=0.005), and availability of mammography services (AOR =1.80, 95% CI: 1.29–2.51, p=0.001). Lower knowledge was associated with having more than 3 children and community stigma (AOR= 0.65, 95% CI: 0.51–0.84, p=0.001).

### Factors associated with HBM attitude variables

As detailed in Supplementary table 1, SA women perceived BC to be less serious than US women, perceived greater benefits for and lower barriers to BSE (though with no differences in mammography screening barriers), greater self-efficacy for BSE and cues to screening action than US women. High knowledge, a family history of BC, being employed, moderate to severe depression/anxiety and BC stigma were all associated with greater perceived susceptibility or seriousness of BC, while high knowledge and stigma also increased the perceived benefit of mammography screening. Older age, tertiary education, and availability of screening services were associated with perceived benefits of mammography screening. Self-efficacy and cues to action reflected the transition from belief to perceived ability to act. They were not driven by knowledge but increased with good health, screening service availability, and stigma. Cues to action was directly associated with older age and higher socioeconomic status (tertiary education, being employed, having less children) and indirectly associated with mental health issues.

### Factors associated with breast cancer screening practices

As per Table 3, SA women showed consistently lower odds of screening uptake across all three screening modalities (ever screened (AOR =0.50, 95% CI 0.39-0.64, p < 0.001), CBE (AOR =0.37, 95% CI 0.28-0.50, p<0.001) and mammography (AOR=0.27, 95% CI 0.21-0.35, p<0.001). Available mammography screening services was the most impactful factor associated with increased screening behavior, (ever screened (AOR=3.53, 95% CI 2.51-4.96), CBE screening (AOR =3.18, 95% CI 2.22-4.54), and mammography screening (AOR=5.64, 95% CI 3.86-8.24), all p< 0.001). Age 55-70 years was also impactful, particularly for mammography screening (AOR=5.70, 95% CI: 4.04-8.05, p<0.001). Higher education and living with a partner also positively associated with screening uptake, as did a family history of BC and stigma. Knowledge was not associated with mammography screening practice, though it did associate with CBE. Perceived susceptibility and seriousness did not associate with screening behavior, but perceived mammography screening benefits, positively impacted screening behavior. Model performance was highest for mammography (Pseudo R^2^ = 0.268), followed by CBE (0.210) and ever screened (0.196).

## Discussion

This binational analysis provides a granular account of why BC screening gaps persist between SA and the USA, even when women report broadly pro-screening beliefs. Three findings stand out. First, SA women had markedly lower screening uptake across all modalities (ever screened, CBE, and mammography), and higher self-efficacy for BSE and cues to action than US women. Second, BC knowledge shaped cognitive appraisals, specifically susceptibility, benefits of mammography, and perceived barriers, but knowledge acquisition did not translate into higher self-efficacy or stronger cues. Third, the strongest and most consistent driver of behavior was structural access: perceived availability

### Screening behavior is primarily constrained by access, not motivation

The magnitude and consistency of service availability leading to screening association supports a central interpretation: in SA, the belief–behavior pathway is frequently interrupted by health-system constraints rather than lack of intention. This aligns with broader evidence that organized screening infrastructure, affordability, geographic access, and service reliability are decisive in determining screening participation, especially for mammography, which is resource-intensive and requires functioning referral pathways and follow-up capacity. The WHO has long emphasized that mammography screening is beneficial primarily when implemented within well-resourced systems that can ensure timely diagnosis, and effective treatment; in lower-resource settings, strengthening early diagnosis pathways (symptom awareness, clinical evaluation, downstaging strategies) is often prioritized over population mammography screening programs. In SA specifically, population-based evidence suggests historically low mammography coverage, reinforcing that the lower uptake observed here is consistent with national patterns[48]. This “access-first” conclusion also reconciles the apparent paradox of SA women reporting higher self-efficacy and cues to action yet lower screening practice: self-efficacy may reflect confidence in personal action (e.g., noticing symptoms, performing BSE) while mammography and CBE require system responsiveness (availability, affordability, and acceptable service interactions). Qualitative work from rural SA confirms service barriers alongside fear and stigma, as proximal determinants of low screening participation[49].

### Knowledge changes risk appraisal, but may not automatically produce action

The modelling clarifies where BC knowledge influences practice. Higher knowledge associated with perceptions of greater perceived susceptibility to BC, benefits of mammography screening, and fewer barriers, consistent with prior SA KAP studies showing that limited knowledge and misconceptions are linked to poor screening-related attitudes and practices [19]. However, motivational constructs of self-efficacy and cues to action were shaped by contextual conditions (health status and service availability) and psychosocial environment (stigma; mental health symptoms). This is an important extension of the literature as much prior work, particularly in LMICs, implicitly treats knowledge deficits as the dominant lever for improving screening. Our data suggests that knowledge though necessary for accurate risk appraisal and understanding of screening benefits, may be insufficient to change behavior when structural and psychosocial constraints remain. That distinction has major intervention implications: education campaigns alone may improve perceived susceptibility and benefits but will underperform if not paired with reachable and reliable screening pathways, affordable, good quality diagnostic and treatment services.

### Psychosocial factors have dual and sometimes counterintuitive roles

The inverse association between stigma and knowledge, coupled with stigma’s positive associations with some belief/behavior outcomes, suggests that stigma may operate through multiple mechanisms. In many settings, stigma reduces open discussion and help-seeking and is consistently reported as a barrier to screening and early presentation [49]. Yet stigma can also increase salience, fear, and perceived social consequences, potentially triggering screening among women who have access and support. Alternatively, it may reflect greater exposure to BC narratives (and thus heightened awareness of stigma) among women already engaged with health services or through family members with BC. However, when poverty precludes access to such services, community fear, stigma toward BC and cultural beliefs deter screening[37, 50]. These possibilities underline the value of treating stigma not as a single barrier construct, but as a multidimensional social process (anticipated judgement, enacted discrimination, internalized shame) that can differentially affect knowledge acquisition versus action. The finding that mild/moderate anxiety or depression correlated with higher knowledge but reduced cues to action is similarly nuanced. One plausible explanation is differential contact with health services and information-seeking among women experiencing distress (raising knowledge), while the same symptoms reduce activation and follow-through (lower cues). This fits wider evidence that social determinants and psychosocial stressors shape screening behavior through competing pathways, including capacity to navigate care and sustain preventive routines[51].

Our findings align with global systematic reviews showing that screening behavior is influenced by sociodemographic factors; the level of social support from family or personal autonomy and health services; knowledge and personal and family health history (including BC); perceptions and attitudes toward BC risk; cultural factors; the availability of screening services; the degrees of information and encouragement for screening uptake provided by healthcare providers, as well as by self-efficacy and cues to action[37, 43, 50, 52]. These reviews confirm that mammography screening is used predominantly in high-income countries, while BSE becomes more important where mammography services are scarce—consistent with SA women perceiving greater benefits from BSE [50, 52]. They further corroborate our finding that self-efficacy and cues to action are strongly associated with screening uptake[37, 50].

On the US side, our findings also sit well within the established literature on screening disparities driven by social determinants (education, employment, support structures) and access-mediated factors such as insurance and service availability. The important point here is that even with more favorable knowledge and access on average, US women in lower-income strata still report structural vulnerabilities (unemployment, poorer perceived health), reinforcing that inequities persist within high-income settings, though the dominant constraints differ in degree and nature.

### Strengths and Limitations

This study draws on a large, cross-sectional sample from SA and the USA, with comparable age distributions and broad representation across household income and living standards. This design enables robust cross-country comparisons of BC knowledge, attitudinal constructs and their progressive influence on BC screening practice. The limitations include that more SA women were from in-country higher income/wealth strata than their US counterparts which may have biased findings. The cross-sectional design prevents causal inference. Screening behavior is self-reported and may be subject to recall and social desirability bias, which could differ by country. Residual confounding is possible, as key factors such as health insurance, type of healthcare provider, and mode of access to services were not fully captured and are relevant in interpreting structural and access-related differences between countries.

### Implications for practice

These findings demonstrate that BC screening engagement is shaped by psychosocial influences, sociodemographic factors and, most decisively, structural access, with clear differences between SA and the US. Knowledge, age, and other sociodemographic factors improved awareness and risk perceptions, but screening uptake requires readily available services. Psychosocial factors such as stigma and poor mental health shaped early attitudes and, in some cases, amplified cues to action, highlighting the need for culturally responsive strategies. The results suggest the need for complementary intervention priorities across contexts. In SA, where structural access remains the primary constraint, strengthening service capacity and community access is a prerequisite. This may include scaling primary health clinic- and community-based CBE screening services for the majority population accessing public health services where expanding mammography screening services is currently cost-prohibitive. In the US, where population-based mammography screening is almost universally accessible, inequities in access for marginalized minority populations require attention. Multi-component approaches that reduce financial and logistical access barriers such as insurance support, while reinforcing cues to action through provider recommendations and reminders, should accompany interventions aimed at raising screening-related knowledge. Across both countries, interventions that address barriers such as fear, anticipated pain, fatalism, and stigma and amplify cues to action through community mobilization and provider engagement may also increase screening uptake. Effective policy and practice must therefore integrate demand-generation strategies with tangible service provision, ensuring equitable access, supporting health literacy, and addressing context-specific constraints: structural expansion in SA versus residual access inequities in the USA.

## Conclusion

This study extends the screening literature by showing that, across SA and US wealth strata, knowledge improves risk appraisal, but BC screening engagement is also shaped by structural access and psychosocial factors. Availability of mammography and CBE emerges as the strongest driver of uptake, while psychosocial influences such as stigma, mental and personal health and knowledge, shape early attitudes that drive motivational cues. Closing these gaps requires integrated strategies that align demand-side approaches: increasing knowledge, reducing barriers and strengthening cues to action, with supply-side solutions, including expanded and equitable access to screening, diagnostic and treatment services.

## Supplementary Material

This is a list of supplementary files associated with this preprint. Click to download.

• Supplementarytable1.xlsx

• AppendixAUSAKAPQuestionnaire.docx

• AppendixBSAKAPQuestionnaire.docx

## Figures and Tables

**Table 1:** Sociodemographic characteristics of participants and their knowledge, attitudes and practice scores for breast cancer and screening

Variables	Total sample		South Africa (SA)		United States of America (US)	
	N	%	N	%	N	%
**Number of women enrolled**	2000	100	1000	50	1000	50
**Age**						
35 - 44 years	400	20.0	200	20.0	200	20.0
45 - 54 years	800	40.0	400	40.0	400	40.0
55 - 70 years	800	40.0	400	40.0	400	40.0
**Education**						
Primary/Secondary	1063	53.2	557	55.7	506	50.6
Tertiary	937	46.8	443	44.3	494	49.4
**Employment**						
Unemployed	785	39.2	311	31.1	474	47.4
Part-time	190	9.5	98	9.8	92	9.2
Full-time	1018	50.9	591	59.1	427	42.7
Prefer not to say	7	0.4	0	0.0	7	0.7
**Income**						
≤$25k	230	23.0	-	-	230	23.0
$25k -<$50k	258	25.8	-	-	258	25.8
$50k-<$75k	181	18.1	-	-	181	18.1
$75k-<$100k	117	11.7	-	-	117	11.7
$100k+	154	15.4	-	-	154	15.4
Prefer not to say	60	6.0	-	-	60	6.0
**Living standard measure (LSM)**						
1-7	336	33.6	336	33.6	-	-
8-10	664	66.4	664	66.4	-	-
**Marital status**						
Single/Never married	496	24.8	287	28.7	209	20.9
Married/Live with partner	972	48.6	420	42.0	552	55.2
Divorced	387	19.3	209	20.9	178	17.8
Widowed	145	7.3	84	8.4	61	6.1
**Parity**						
No children	452	22.6	121	12.1	331	33.1
1-2	1019	50.9	558	55.8	461	46.1
3+	529	26.5	321	32.1	208	20.8
**Covariates**						
**Family history of breast cancer**						
No/Don’t know	1414	70.7	726	72.6	688	68.8
Yes	586	29.3	274	27.4	312	31.2
**Perceived health status (0-100)**						
Mean (SD)	70.3 (21.1)	-	74.5 (19.8)	-	66.1 (21.4)	-
Median (IQR)	75 (54 – 86)	-	80 (61 – 90)	-	70 (50 – 83)	-
Low (0-44)	249	12.5	83	8.3	166	16.6
Moderate (45-87)	1277	63.8	606	60.6	671	67.1
High (88-100)	474	23.7	311	31.1	163	16.3
**Perceived mental health symptoms**						
Not anxious or depressed	806	40.3	431	43.1	806	40.3
Slightly/moderately anxious or depressed	1027	51.3	510	51.0	1027	51.3
Severely/extremely anxious or depressed	167	8.4	59	5.9	167	8.4
**Clinical breast examination available**						
No	416	20.8	221	22.1	195	19.5
Yes	1584	79.2	779	77.9	805	80.5
**Mammography available**						
No	420	21.0	263	26.3	157	15.7
Yes	1580	79.0	737	73.7	843	84.3
**Perceived stigma around breast cancer**						
No/Don’t know	1600	80.0	736	73.6	864	86.4
Yes	400	20.0	264	26.4	136	13.6
**Knowledge, attitude and behavior variables**						
**Breast cancer knowledge score (3-12)**						
Low (1-9)	299	14.9	209	20.9	90	9.0
Moderate (10)	1428	71.4	703	70.3	725	72.5
High (11-12)	273	13.7	88	8.8	185	18.5
**Health Belief Model attitude variables**						
**Perceived susceptibility to breast cancer score (5-25)**						
Low (5-10)	482	24.1	276	27.6	206	20.6
Moderate (11-16)	828	41.4	412	41.2	416	41.6
High (17-25)	690	34.5	312	31.2	378	37.8
**Perceived seriousness of breast cancer score (7-35)**						
Low (5-15)	449	22.5	253	25.3	196	19.6
Moderate (16-26)	1019	51.4	490	49.0	539	53.9
High (27-35)	522	26.1	257	25.7	265	26.5
**Perceived benefits of breast self-examination score (6-30)**						
Low (6–14)	44	2.20	16	1.60	28	2.80
Moderate (15–22)	1,070	53.50	459	45.90	611	61.10
High (23–30)	886	44.30	525	52.50	361	36.10
**Perceived benefits of mammography score (6-30)**						
Low (6–14)	43	2.15	22	2.20	21	2.10
Moderate (15–22)	913	45.65	449	44.90	464	46.40
High (23–30)	1,044	52.20	529	52.90	515	51.50
**Perceived barriers to breast self-examination score (6-30)**						
Low (6–14)	1,537	76.85	805	80.50	732	73.20
Moderate (15–22)	417	20.85	180	18.00	237	23.70
High (23–30)	46	2.30	15	1.50	31	3.10
**Perceived barriers to mammography screening score (5-25)**						
Low (5–11.7)	1,114	55.70	559	55.90	555	55.50
Moderate (11.8–18.4)	778	38.90	392	39.20	386	38.60
High (18.5–25)	108	5.40	49	4.90	59	5.90
**Perceived self-efficacy for breast self-examination score (11-55)**						
Low (11-30)	564	28.2	240	24.0	324	32.4
Moderate (31-47)	979	48.9	510	51.0	469	46.9
High (48-55)	457	22.9	250	25.0	207	20.7
**Perceived cues to action (for breast cancer screening) score (7-35)**						
Low (7-22)	473	23.6	194	19.4	279	27.9
Moderate (23-32)	943	47.2	476	47.6	467	46.7
High (33-35)	584	29.2	330	33.0	254	25.4
**Screening practice prevalence**						
Ever screened (yes)	1373	69	616	62	757	76
Ever had a clinical breast examination (yes)	1585	79	711	71	874	87
Ever had a screening mammography (yes)	1292	65	509	51	783	78

KAP= Knowledge, Attitudes and Practice for breast cancer screening; IQR=interquartile range

**Table 2.** Generalized ordered logistic regression analysis of factors associated with high breast cancer knowledge among 2000 women enrolled from the US and SA

Variables	Adjusted OR	95% CI	p-value
**Country**			
USA	1 (ref)		
South Africa	0.50	0.40–0.63	<0.001
**Age group**			
35-44	1 (ref)		
45-54	1.08	0.82–1.41	0.60
55-70	1.06	0.79–1.41	0.71
**Education**			
Primary/Secondary	1 (ref)		
Tertiary	1.32	1.08–1.62	0.008
**Employment**			
Unemployed	1 (ref)		
Part-time	0.92	0.65–1.32	0.67
Full-time	0.84	0.67–1.05	0.12
**Marital status**			
Single	1 (ref)		
Living with partner	1.23	0.96–1.58	0.10
Divorced	1.46	1.08–1.99	0.015
Widowed	1.22	0.80–1.85	0.36
**Parity**			
None	1 (ref)		
1-2 children	0.84	0.65–1.10	0.20
3+ children	0.69	0.51–0.93	0.015
**Family history of breast cancer**			
No	1 (ref)		
Yes	1.08	0.87–1.34	0.47
**Self-rated health status**			
Low	1 (ref)		
Moderate	0.96	0.71–1.32	0.82
High	0.81	0.56–1.17	0.26
**Mental health issues**			
None	1 (ref)		
Slightly/moderately affected	1.36	1.10–1.69	0.005
Severely affected	1.03	0.69–1.53	0.88
**Service availability - CBE**			
Not available	1 (ref)		
Available	1.04	0.75–1.45	0.81
**Service availability - Mammography**			
Not available	1 (ref)		
Available	1.80	1.29–2.51	0.001
**Stigma**			
No	1 (ref)		
Yes	0.65	0.51–0.84	0.001

Odds ratios are adjusted for all other variables in the model. Proportional odds assumption was met for all covariates except stigma.

CBE= Clinical Breast Examination

**Table 3.** Multivariable logistic regression analysis of factors associated with ever having been screened for breast cancer; ever having received a clinical breast examination; ever receiving mammography screening (n=2000)

	Ever Screened			Clinical Breast Examination			Mammography		
Variables	AOR	95% CI	p-value	AOR	95% CI	p-value	AOR	95% CI	p-value
**Country**									
USA	1 (ref)			1 (ref)			1 (ref)		
South Africa	**0.50**	**0.39–0.64**	**<0.001**	**0.37**	**0.28–0.50**	**<0.001**	**0.27**	**0.21–0.35**	**<0.001**
**Age group**									
35-44	1 (ref)			1 (ref)			1 (ref)		
45-54	**1.42**	**1.06–1.91**	**0.017**	1.19	0.86–1.65	0.283	**2.18**	**1.62–2.94**	**<0.001**
55-70	**2.20**	**1.59–3.05**	**<0.001**	**1.75**	**1.21–2.53**	**0.003**	**5.70**	**4.04–8.05**	**<0.001**
**Education**									
Primary/Secondary	1 (ref)			1 (ref)			1 (ref)		
Tertiary	**1.45**	**1.15–1.82**	**0.002**	**1.62**	**1.24–2.11**	**<0.001**	**1.51**	**1.19–1.92**	**0.001**
**Employment**									
Unemployed	1 (ref)			1 (ref)			1 (ref)		
Part-time	0,97	0.65–1.44	0.879	0.80	0.51–1.25	0.327	0.99	0.65–1.52	0.975
Full-time	1.07	0.83–1.38	0.581	0.83	0.62–1.11	0.211	0.91	0.70–1.18	0.467
Prefer not to answer	4.42	0.44–44.59	0.208	0.44	0.07–2.85	0.391	2.97	0.29–30.36	0.358
**Marital status**									
Single	1 (ref)			1 (ref)			1 (ref)		
Living with partner	1.19	0.90–1.56	0.226	1.54	1.14–2.09	0.005	**1.70**	**1.28–2.26**	**<0.001**
Divorced	**1.43**	**1.01–2.02**	**0.046**	**1.76**	**1.19–2.61**	**0.005**	**1.53**	**1.08–2.18**	**0.017**
Widowed	1.31	0.81–2.13	0.276	**2.00**	**1.12–3.59**	**0.020**	**1.36**	**0.83–2.22**	**0.219**
**Parity**									
None	1 (ref)			1 (ref)			1 (ref)		
1-2 children	1.29	0.96–1.73	0.098	1.30	0.92–1.83	0.132	1.13	0.83–1.55	0.439
3+ children	1.32	0.94–1.84	0.108	1.10	0.75–1.61	0.627	0.87	0.61–1.23	0.424
**Stigma**									
No	1 (ref)			1 (ref)			1 (ref)		
Yes	**1.50**	**1.11–2.01**	**0.007**	1.14	0.83–1.58	0.415	**1.55**	**1.16–2.09**	**0.004**
**Family history of breast cancer**									
No	1 (ref)			1 (ref)			1 (ref)		
Yes	**1.42**	**1.10–1.82**	**0.007**	**1.46**	**1.08–1.96**	**0.012**	**1.52**	**1.18–1.97**	**0.001**
**Self-rated health status**									
Low	1 (ref)			1 (ref)			1 (ref)		
Moderate	1.08	0.76–1.52	0.682	1.05	0.70–1.57	0.801	1.08	0.75–1.54	0.690
High	0.89	0.59–1.34	0.590	0.75	0.48–1.19	0.227	0.87	0.57–1.33	0.519
**Mental health issues**									
None	1 (ref)			1 (ref)			1 (ref)		
Slightly/moderately affected	1.15	0.90–1.47	0.252	1.06	0.80–1.41	0.667	1.07	0.83–1.37	0.623
Severely affected	1.10	0.71–1.70	0.668	0.97	0.59–1.59	0.908	1.54	0.97–2.43	0.067
**Screening service availability**									
CBE available	**1.41**	**1.00–1.99**	**0.053**	**1.54**	**1.06–2.22**	**0.023**	0.94	0.64–1.38	0.749
Mammography available	**3.53**	**2.51–4.96**	**<0.001**	**3.18**	**2.22–4.54**	**<0.001**	**5.64**	**3.86–8.24**	**<0.001**
**KNOWLEDGE of BC and symptoms**									
Low	1 (ref)			1 (ref)			1 (ref)		
Moderate	1,33	0.98–1.81	0.063	1.58	1.15–2.18	0.005	1.04	0.76–1.42	0.812
High	**2.18**	**1.39–3.43**	**0.001**	**2.16**	**1.30–3.60**	**0.003**	1.38	0.88–2.18	0.162
**ATTITUDES: Perceived Susceptibility to BC**									
Low	1 (ref)			1 (ref)			1 (ref)		
Moderate	1.18	0.89–1.57	0.25	0.99	0.72–1.36	0.94	1.24	0.93–1.66	0.15
High	1.14	0.83–1.55	0.42	1.16	0.81–1.64	0.42	**1.37**	**1.00–1.88**	**0.05**
**Perceived Seriousness of BC**									
Low	1 (ref)			1 (ref)			1 (ref)		
Moderate	1.10	0.82–1.47	0.52	**1.64**	**1.18–2.28**	**0.003**	1.04	0.77–1.39	0.802
High	1.00	0.71–1.40	0.99	1.14	0.79–1.66	0.482	1.08	0.76–1.52	0.679
**Breast Self-examination benefits**									
Low	1 (ref)			1 (ref)			1 (ref)		
Moderate	1.07	0.52–2.21	0.86	1.27	0.58–2.78	0.546	0.72	0.33–1.57	0.405
High	1.11	0.52–2.36	0.78	1.01	0.45–2.27	0.988	0.51	0.23–1.13	0.098
**Mammography screening benefits**									
Low	1 (ref)			1 (ref)			1 (ref)		
Moderate	1.37	0.67–2.79	0.39	0.93	0.41–2.08	0.855	1.47	0.69–3.13	0.324
High	1.85	0.89–3.83	0.097	1.04	0.45–2.36	0.934	**2.95**	**1.36–6.40**	**0.006**
**BSE barriers**									
Low	1 (ref)			1 (ref)			1 (ref)		
Moderate	1.07	0.79–1.44	0.67	0.81	0.58–1.12	0.197	1.17	0.85–1.60	0.335
High	1.23	0.53–2.85	0.64	0.64	0.27–1.54	0.321	0.72	0.30–1.75	0.470
**Mammography screening barriers**									
Low	1 (ref)			1 (ref)			1 (ref)		
Moderate	**0.55**	**0.43–0.71**	**<0.001**	**0.67**	**0.50–0.90**	**0.008**	0.65	0.50–0.84	0.001
High	**0.48**	**0.28–0.84**	**0.010**	0.75	0.41–1.36	0.340	0.64	0.36–1.14	0.128
**Self-efficacy in BSE**									
Low	1 (ref)			1 (ref)			1 (ref)		
Moderate	0.92	0.70–1.21	0.57	1.26	0.93–1.71	0.135	0.92	0.69–1.23	0.575
High	0.85	0.60–1.22	0.38	1.41	0.94–2.12	0.097	0.83	0.57–1.19	0.302
**Cues to action**									
Low	1 (ref)			1 (ref)			1 (ref)		
Moderate	1.29	0.98–1.70	0.073	1.12	0.81–1.54	0.491	1.36	1.02–1.83	0.039
High	**1.90**	**1.33–2.71**	**<0.001**	1.54	1.02–2.32	0.039	1.58	1.09–2.27	0.015

Abbreviations: AOR= Adjusted odds ratio, CBE= Clinical Breast Examination; BC= Breast cancer; BSE = Breast Self-examination; Ref = reference.

All models adjusted for variables shown.

## Data Availability

All data and materials are made available within the paper and supporting files. The authors confirm that de-identified participant data will be made available to all interested researchers upon reasonable request by contacting the corresponding author
